# Two Decades of Progress in Personalized Medicine of Colorectal Cancer in Serbia—Insights from the Institute for Oncology and Radiology of Serbia

**DOI:** 10.3390/biomedicines12102278

**Published:** 2024-10-08

**Authors:** Milena Cavic, Neda Nikolic, Mladen Marinkovic, Ana Damjanovic, Ana Krivokuca, Miljana Tanic, Marko Radulovic, Aleksandra Stanojevic, Luka Pejnovic, Marija Djordjic Crnogorac, Ana Djuric, Miodrag Vukovic, Vanja Stevanovic, Jelena Kijac, Valentina Karadzic, Srdjan Nikolic, Suzana Stojanovic-Rundic, Radmila Jankovic, Jelena Spasic

**Affiliations:** 1 Department of Experimental Oncology, Institute for Oncology and Radiology of Serbia, 11000 Belgrade, Serbia; zaanu011@gmail.com (A.D.); tanic.miljana@ncrc.ac.rs (M.T.); marko@radulovic.net (M.R.); astefanovic496@gmail.com (A.S.); ana.djuric@ncrc.ac.rs (A.D.); miodragvuk97@gmail.com (M.V.); vanjastevanovic999@gmail.com (V.S.); jelenakijac12@gmail.com (J.K.); jankovicr@ncrc.ac.rs (R.J.); 2 Clinic for Medical Oncology, Institute for Oncology and Radiology of Serbia, 11000 Belgrade, Serbia; neda.nikolic@ncrc.ac.rs; 3 Clinic for Radiation Oncology and Diagnostics, Department of Radiation Oncology, Institute for Oncology and Radiology of Serbia, 11000 Belgrade, Serbia; mladen309@gmail.com (M.M.); stojanovics@ncrc.ac.rs (S.S.-R.); 4 Faculty of Medicine, University of Belgrade, 11000 Belgrade, Serbia; onkosurge1@yahoo.com; 5 Genetic Counseling for Hereditary Cancers Department, Institute for Oncology and Radiology of Serbia, 11000 Belgrade, Serbia; krivokuca.ana@gmail.com (A.K.); marijadjordjic@gmail.com (M.D.C.); valentina.karadzic@gmail.com (V.K.); 6 Cancer Biology Department, University College London Cancer Institute, London WC1E 6DD, UK; 7 Clinic for Surgical Oncology Institute for Oncology and Radiology of Serbia, 11000 Belgrade, Serbia; lpejnovic14@gmail.com

**Keywords:** colon cancer, biomarker, pharmacogenomics, precision medicine, rectal cancer

## Abstract

Background: It is projected that, by 2040, the number of new cases of colorectal cancer (CRC) will increase to 3.2 million, and the number of deaths to 1.6 million, highlighting the need for prevention strategies, early detection and adequate follow-up. In this study, we aimed to provide an overview of the progress in personalized medicine of CRC in Serbia, with results and insights from the Institute for Oncology and Radiology of Serbia (IORS), and to propose guidance for tackling observed challenges in the future. Methods: Epidemiological data were derived from official global and national cancer registries and IORS electronic medical records. Germline genetic testing for Lynch syndrome was performed by Next Generation Sequencing. *RAS* and *BRAF* mutation analyses were performed using qPCR diagnostic kits. Results: Epidemiology and risk factors, prevention and early detection programs, as well as treatment options and scientific advances have been described in detail. Out of 103 patients who underwent germline testing for Lynch syndrome, 19 (18.4%) showed a mutation in MMR genes with pathogenic or likely pathogenic significance and 8 (7.8%) in other CRC-associated genes (*APC*, *CHEK2*, *MUTYH*). Of 6369 tested patients, 50.43% had a mutation in *KRAS* or *NRAS* genes, while 9.54% had the V600 mutation in the BRAF gene. Conclusions: Although significant improvements in CRC management have occurred globally in recent years, a strategic approach leading to population-based systemic solutions is required. The high incidence of young-onset CRC and the growing elderly population due to a rise in life expectancy will be especially important factors for countries with limited healthcare resources like Serbia.

## 1. Introduction

The global burden of colorectal cancer (CRC) in 2022 was reported as over 1.9 million newly diagnosed cases (9.6% of total, rank 3) and over 900,000 deaths (9.6 of total, rank 2), making it one of the most prominent cancer diseases [1]. Projections of increases up to 3.2 million newly diagnosed and 1.6 million mortality cases by 2040 [2] highlight the necessity for prevention strategies of affecting modifiable risk factors, coupled with early detection and adequate follow up of premalignant lesions. Screening colonoscopy is likely to have a much stronger protective effect which manifests much earlier than recently reported. With increasing rates in incidence (1.7% per year) and mortality (0.5% per year) of colon, rectum and anus carcinomas in the young adult population (15–49 years of age),more efficient approaches and spread of health equity plans are paramount [3]. The financial burden of CRC in Europe was assessed at approximately 19 billion EUR in 2023, with an expected increase in the coming years due to unhealthy lifestyles. Globally in 2019, CRC accounted for over 24 million Disability Adjusted Life Years (DALYs), with low- and middle-income regions comprising approximately 75% of all global DALYs [4].

CRC remains a significant public health issue in Serbia. The morbidity and mortality induced by colorectal, lung, breast, stomach and cervical cancers had a substantial population-wise impact in the period from 1999 to 2003, inducing 73,197 (19.9/1000 population) DALYs in men and 60,482 (15.6/1000) DALYs in women, annually [5]. The most recent epidemiological data available is from 2021, showing that the age-standardized incidence rates (ASR) for CRC in Serbia were 44.3 and 24.7 males and females, respectively. The ASR has shown a steady increase from 1990 to 2021, rising from 20 to 44.3 in males and from 18 to 24.7 in females [6]. Analysis of cancer trends in the Balkans in the period from 1990 to 2019, has shown that years of life lost (YLL) rates as a consequence of CRC are forecasted to increase by 2030 [7]. Described country-specific barriers are mostly related to lifestyles and low uptake of screening and early detection, as well as availability of treatment options. Most expenses due to CRC pertain to advance stage disease inpatient care, targeted therapies, diagnostic imaging and invasive radiology procedures [8]. Although in line with the rise in CRC incidence, the total cost of accompanying medical services is even higher (27% increase in the period 2014 to 2017). 

The purpose of this study was to analyze the progress in personalized approaches for early detection, clinical and molecular diagnostics and treatment strategies as well as the progress in molecular and diagnostic techniques in the management of individuals with CRC in Serbia. The results were focused on insights from the Institute for Oncology and Radiology of Serbia (IORS) and the National Cancer Research Center, with the additional aim to propose guidance for tackling observed challenges in the future.

## 2. Material and Methods

Epidemiological data were derived from official global and national cancer registries.

The data had been collected from all Serbian healthcare institutions and imported into the population-based national registry performed by the Institute for Public Health “Dr Milan Jovanovic Batut”. A cancer registry search was performed to acquire data on patient demographics, tumor characteristics, stage of disease, treatment information and outcomes, where applicable. The search strategy was focused on determining cancer patterns among various sub-populations (sex, age) and monitoring cancer trends over time, in an effort to propose future strategies for advancing clinical, epidemiological and health services research. Annual patient numbers treated at IORS were derived from electronic medical records. Diagnostic analyses were conducted using formalin-fixed paraffin-embedded tissue samples (FFPE), extracting DNA using either the QIAamp DNA FFPE Tissue Kit (Qiagen, Manchester, UK) or the Cobas^®^ DNA Sample Preparation Kit (Roche Molecular Diagnostics, Mannheim, Germany). RAS mutation analysis was conducted using various in vitro diagnostics (IVD)-registered methods, including the KRAS StripAssay, TheraScreen^®^ K-RAS Mutation Kit on the Applied Biosystems 7500 Real-Time PCR, TheraScreen^®^ KRAS RGQ PCR Kit on the Qiagen Rotor Gene Q, Cobas^®^ KRAS Mutation Test on the Cobas^®^ 4800, and AmoyDx KRAS/NRAS Mutation Detection Kit on the Applied Biosystems 7500 Real-Time PCR or LightCycler^®^ 480 Instrument II. Graphical schemes were reported according to the PRISMA 2020 guidelines [9]. Statistical analysis included determination of frequency, percentage, mean/median, standard deviation and range using descriptive methods in GraphPad Prism (V.9, GraphPad Software, La Jolla, CA, USA).

All clinical and molecular testing and research analyses were approved by the Ethics Committee of the Institute for Oncology and Radiology of Serbia (Approval No. 01-1/2023/701 from 30 March 2023). Experiments were performed according to guidelines issued in the Helsinki Declaration of 1975, as revised in 2013. All study participants signed informed consent. 

## 3. Results

### 3.1. Epidemiology and Risk Factors in Serbia

**Figure 1:**
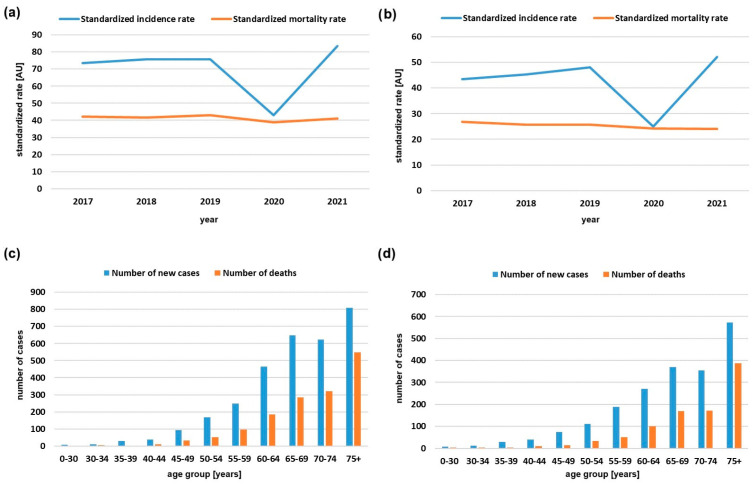
Standardized incidence and mortality rates in a 5-year period (2017–2021) in (**a**) males and (**b**) females. Number of new cases and deaths in (**c**) males and (**d**) females per age group in 2021. Data derived from official reports of the Serbian Cancer Registry of the Institute of Public Health of Serbia “Dr Milan Jovanović Batut” [6].

The standardized incidence rates of CRC in central Serbia have been steadily increasing, by about 0.7% per year for females and 1% per year for males, in the period between 1999 and 2020 [10]. According to data published by the Serbian Cancer Registry of the Institute of Public Health of Serbia “Dr Milan Jovanović Batut” in 2021, CRC was newly diagnosed in 5174 patients (2029 females, 3145 males), comprising 12.4% of all cancers, leading to its ranking as the second most signficant cause of cancer-related morbidity and mortality [6]. By incidence, only lung cancer was more common than CRC among males (14.1%), and among females it was ranked behind breast and lung cancer (10.5%). Analysis of standardized rates of incidence and mortality and newly diagnosed cases in males and females per age group in a 5-year period (2017–2021) obtained from the Serbian Cancer Registry are presented in Figure 1. As in other countries, incidence of young-onset CRC has been gradually on the rise in the last decade [11]. 

In general, risk factors for developing CRC can be divided into modifiable and non-modifiable. Risk factors which can be modified are mostly connected to lifestyle, such as alcohol consumption, smoking, diet high in processed foods and fat and low in fiber, obesity and sedentary lifestyle. Risk factors which are not modifiable are age, race, sex at birth, history of colorectal polyps/cancer, inflammatory bowel disease, personal history of irradiation of abdomen or pelvis, family history of adenomatous polyps and CRC and certain genetic syndromes [12]. In Serbia, the combined effect of a red-meat based diet, and a high smoking rate have been suggested as predominant CRC risk factors, although a relevant population-based study with high statistical power has not been performed. 

### 3.2. Screening, Prevention and Early Detection Programs 

Primary (avoiding risk factors and promoting protective factors) and secondary prevention (screening) of CRC have been proven to decrease CRC incidence and mortality. Organized CRC screening was implemented in Serbia in December of 2012, according to guidelines for the National screening program for CRC in Serbia, which have been made on the basis of the Council of the European Union Recommendations for cancer screening of 2003 (https://eur-lex.europa.eu/eli/reco/2003/878/oj, accessed on 3 October 2024). All individuals older than 50 are offered fecal occult blood test (FOB), and those with a positive finding are referred to colonoscopy. The program is carried out through the primary care physicians initially, and if the FOB test is positive, subjects are referred to secondary centers. Between 2013 and 2018, three two-year cycles of CRC screening were carried out. During that time, on average, 52% of individuals responded to screening invitations. The FOB test was positive in 7.2% and of those, 43.3% agreed to a colonoscopy. Furthermore, colonoscopy detected polyps in 39% of all individuals who agreed to a colonoscopy, while carcinoma was histologically confirmed in 0.2%. The positive predictive value was 27.1% for adenoma and 14.6% for carcinoma [13]. Program for improved control of cancer in the Republic of Serbia in 2020–2022 (http://demo.paragraf.rs/demo/combined/Old/t/t2020_08/SG_105_2020_001.htm, accessed on 3 October 2024) included the preparation of an Action plan to be implemented population wise [14]. Evaluation of the first results of this initiative is pending.

### 3.3. Hereditary Risk Factors and Molecular Testing for Lynch Syndrome

Lynch syndrome, or hereditary nonpolyposis CRC syndrome (HNPCC), is ranked as number 1 of all hereditary CRC syndromes, with an approximate population prevalence of 2–3%. This inherited condition significantly increases the risk of developing CRC as well as endometrial, ovarian, brain, upper urinary tract, stomach, small intestine, liver, biliary tract and skin cancers. Germline mutations in genes DNA mismatch repair (MMR) genes *MLH1*, *MSH2*, *MSH6*, *EPCAM* and *PMS2* lead to the development of this syndrome [15,16]. The risk varies depending both on the specific gene mutation and sex at birth. Mutations in the *MLH1* and *MSH2* genes have the highest penetrance, with an estimated lifetime chance of CRC at around 50% by age 70. Individuals carrying *MSH6* and *PMS2* mutations have a lower, but still significant risk, particularly *MSH6* carriers, where the lifetime risk is estimated at about 30% by age 70. Additionally, the risk of endometrial cancer is notably high among carriers of MMR gene mutations, especially for those with *MLH1* and *MSH2* mutations, where it is estimated to be between 40 and 60% by age 70 [17]. Recent studies underscore the need for CRC surveillance strategies tailored to the specific MMR gene mutations in Lynch syndrome patients. Intensive surveillance is advised for individuals carrying *MLH1* and *MSH2* mutations, beginning at 25 years of age at 1–2-year intervals. However, for *MSH6* and *PMS2* carriers, starting later and extending the intervals between screenings may be more effective considering the balance between cost and timely tumor detection [18]. According to the European Hereditary Tumour Group (EHTG) and the European Society of Coloproctology (ESCP) guidelines, the prevalence of pathogenic MMR gene carriers is estimated to be approximately one in 300, which equates to around 2.5 million people in Europe [19]. 

Since 2018, The Institute of Oncology and Radiology of Serbia has been conducting germline genetic testing for Lynch syndrome using Next Generation Sequencing technology (TruSight Cancer Panel, Ilumina, Munich, Germany). Patients are selected based on early-onset cancer and family history, following the Amsterdam Criteria and Bethesda Guidelines [20]. In addition to indication of strong family history, patients are also referred for MMR germline genetic testing by tumor boards due to abnormal protein expression results identified through immunohistochemistry. Since the start of MMR germline genetic testing at IORS in 2018 through July 2024, a total of 122 individuals suspected of having HNPCC were referred to the Genetic Counseling for Hereditary Cancers Department at IORS by Serbian medical institutions. Of these, 113 met the Amsterdam/Bethesda criteria for Lynch syndrome testing, and 103 underwent testing so far. All participants provided informed consent for genetic testing, and the testing was performed according to approval of the IORS Ethics Committee. The Serbian Health Insurance Fund reimburses the expenses needed for NGS testing for individuals who meet the national criteria. At the time of this manuscript preparation, IORS was the only national center offering genetic counseling and analysis for HNPCC.

**Figure 2:**
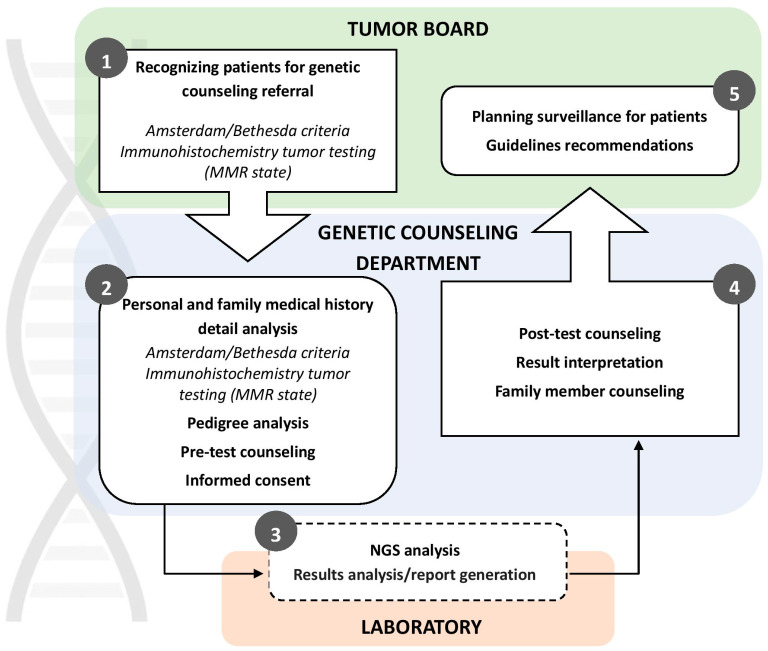
A schematic representation of the genetic testing pathway at the Genetic Counseling for Hereditary Cancers Department of the Institute for Oncology and Radiology of Serbia, the national center for hereditary cancer genetic testing in Serbia.

Pre-test genetic counseling is dedicated to collecting personal and family histories (FH). The probabilities of carrying mutations (CP) were determined using MMRpro (CaGENE 5.2 software, Cancer-Gene, Dallas, TX, USA, http://www4.utsouthwestern.edu/breasthealth/cagene, accessed on 3 October 2024), MMRPredict [21] and PREMM [22] prediction models. Those who did not meet the testing criteria included those with polyposis conditions, clinically diagnosed familial adenomatous polyposis (FAP) or those assessed by prediction models as having a very low risk of Lynch syndrome. Out of the 103 individuals tested, 19 (18.4%) were found to have a pathogenic or likely pathogenic mutation in MMR genes. Pathogenic or likely pathogenic mutations in other CRC-associated genes (*APC*, *CHEK2*, *MUTYH*) were identified in 8 (7.8%) individuals, and variants of unknown significance (VUS) in CRC-related genes (MMR genes, *APC*, *CHEK2*) were detected in 8 (7.8%) individuals. 69 (67%) were wild type (WT) and had no genetic variants in the investigated panel. Regardless of the test results, all patients were invited for post-test genetic counseling at IORS, where further details regarding their families and potential additional testing were discussed. Afterward, patients were re-referred to their medical oncologist to discuss further treatment options (Figure 2). 

The introduction of germline MMR genetic testing at IORS represents a significant advancement in identifying persons and families at risk for Lynch syndrome-associated cancers in Serbia. By implementing genetic testing and counseling services, healthcare professionals can provide personalized screening and preventive measures, potentially alleviating the burden of hereditary cancers within the population. This effort will also aid in identifying the frequency and spectrum of MMR gene mutations within this Slavic population, addressing the knowledge gap about the prevalence of Lynch syndrome genes in this part of Europe. 

### 3.4. Diagnosis and Treatment

#### 3.4.1. Diagnostic Considerations

A patient’s journey towards CRC diagnosis starts at a primary care level, which represents the first contact a person makes with healthcare providers (HCP) upon noticing symptoms. A fecal blood test is offered, and in the case of a positive result, the person is referred to a secondary or tertiary center for further diagnostic procedures [23,24]. Overall, diagnostic capabilities in Serbia have improved, largely due to the widespread availability of endoscopic devices, computed tomography (CT) and magnetic resonance imaging (MRI). Still, the waiting time for radiologic procedures and colonoscopy is still more than 3 months in some areas, and pathology remains an issue. All this leads to an increase in the number of new diagnoses of CRC in advanced stages (~35% metastatic disease) [11,25]. The other problem is the low uptake of screening, which has been available in Serbia for the past ten years, as previously explained. Improvement of the patient journey is an urgent necessity, as around half of patients experience at least a month-long delay to begin treatment [26]. 

#### 3.4.2. Systemic Therapy Approaches 

The introduction of systemic therapies, including chemo, targeted and immunotherapy, has significantly transformed the treatment landscape for advanced-stage CRC [27]. In Serbia, systemic therapy is provided across 5 tertiary centers and around 40 general hospitals for around 2000–3000 patients with metastatic CRC per year. At IORS, around 500 patients in various stages of disease are treated yearly. Systemic therapy has traditionally relied on doublet chemotherapy (Oxalipatin and Irinotecan-based), which were introduced in Serbia in the 2000s. Still, adjuvant use of oxaliplatin-based doublets was not reimbursed until 2020. In systemic approach patient characteristics, sidedness of the tumor and molecular profile are crucial factors in treatment decision-making process. The identification of RAS and BRAF mutations has become crucial in determining the most appropriate systemic treatments. Serbia is a country with long standing drug availability problems. Targeted therapies (Bevacizumab, Cetuximab, Panitumumab) became reimbursed as first-line treatments in 2022, after years of third-line and limited first-line use. By 2010, only 20% of patients received targeted therapy as a first-line treatment [11]. Calculated 5-year overall survival in Serbian patients with CRC is going from 6.5% (95%CI) up to 14% in young-onset CRC (95%CI) [11,25]. 

**Figure 3:**
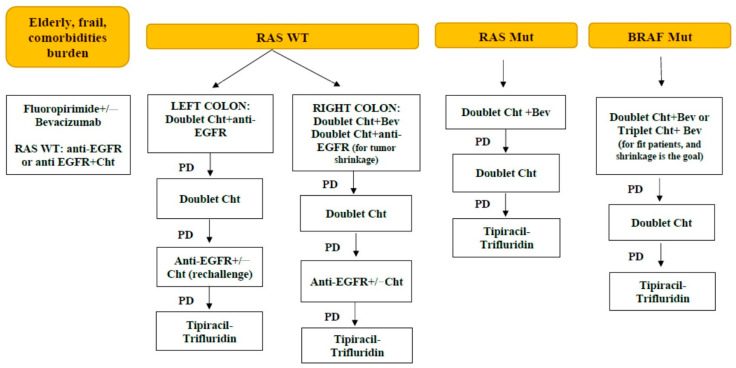
A schematic representation of stage IV unresectable CRC management in Serbia, according to availability of drugs reimbursed by the National Health Insurance Fund. Abbreviation: ChT: chemotherapy; EGFR: epidermal growth factor receptor; PD: progressive disease, Bev: Bevacizumab.

The current management protocol of stage IV unresectable CRC in Serbia is presented in Figure 3. 

Progress in the availability of drugs has been slow, hindered by the limited list of reimbursed medications covered by health insurance. A recent addition is Tipiracil-Trifluridine, available from May 2024, for third-line and beyond treatment. Checkpoint inhibitors (CPI) are still not reimbursed for patients with MSI-high CRC, nor are other EMA and FDA-approved drugs (Regorafenib, Aflibercept, Encorafenib). On the other hand, an area that has seen significant improvement over the past two decades is supportive care, which is currently an integral part of treatment in Serbia, especially at IORS, and is offered upfront, during the active treatment and as end-of-life care.

#### 3.4.3. Radiation Approaches 

Radiotherapy plays a significant role in the multidisciplinary approach to CRC, particularly in locally advanced rectal cancer (LARC). Since the establishment of the radiation oncology residency program in Serbia in 2011, significant progress has been made in training and educating professionals in this field. To further enhance the skills of radiotherapy technicians, a Master’s program for radiation therapists was introduced in 2022. Additionally, residency programs for medical physicists, established in 2013, have contributed to the advancement of offered radiotherapy services.

There are eight radiotherapy centers in Serbia equipped with modern linear accelerators (LINACs), employing three-dimensional conformal radiotherapy (3D-CRT), volumetric modulated arc therapy (VMAT), intensity-modulated radiation therapy (IMRT), stereotactic body radiotherapy (SBRT) and respiratory gating. Stereotactic radiosurgery for brain metastases using gamma knife is available in one center. Image guidance (cone-beam CT included) is available across all centers for treatment delivery. 

The Institute of Oncology and Radiology of Serbia has a long tradition in the treatment of LARC dating back to the early 1990s and was part of the EORTC 22921 study [28], which laid the foundation for the introduction of the neoadjuvant approach in LARC treatment. Each year, approximately 150 patients with CRC undergo radiotherapy at IORS. Most CRC patients requiring radiotherapy have LARC and are treated with long-course neoadjuvant chemoradiotherapy (CRT). Modern treatment protocols in Serbia now involve precise disease staging through MRI as part of the initial evaluation process, as well as novel radiotherapy planning in accordance with international guidelines [29]. Advanced radiotherapy techniques, such as VMAT and IMRT, are widely used. The typical radiotherapy doses range from 50.4 Gy to 54 Gy, applied using either sequential or simultaneous boost techniques in a long-course regimen.

For high-risk patients (clinical T4a/T4b stage, presence of extramural vascular invasion (EMVI), clinical N2 stage, involved circumferential resection margin (CRM), or enlarged lateral lymph nodes), total neoadjuvant therapy (TNT) is often employed. Additional cycles of chemotherapy may be administered before CRT (induction approach) or between the completion of CRT and surgery (consolidation approach) [30,31]. The consolidation approach is usually applied for distally located high-risk rectal cancers, while for larger, more proximally located tumors, we prioritize the induction approach. Routine determination of clinical response is performed 6–8 weeks upon end of neoadjuvant treatment, using digital rectal examination (DRE), rigid proctoscopy and pelvic MRI. For patients with a complete clinical response (cCR) after initial treatment, particularly those with distally located tumors, a ‘watch-and-wait’ approach is now a viable option in Serbia. The neoadjuvant CRT strategy is also utilized in treating local disease recurrence following surgery. For metastatic CRC, conventional radiotherapy and stereotactic techniques are applied, particularly for metastases to the brain, bone and liver, in conjunction with advanced systemic therapies.

#### 3.4.4. Surgical Approaches

Although advanced surgical approaches are available at IORS, challenges in the treatment of CRC include late diagnosis of the disease, which can limit the effectiveness of surgical procedures. The decision on any type of treatment is made in a multidisciplinary manner, taking into account the disease and patient characteristics, and in accordance with relevant clinical practice guidelines [32]. Standard surgical techniques include resection of the affected colon/rectum part with removal of locoregional lymph nodes. Around 350 such surgeries are performed annually at the Clinic for Oncological Surgery of IORS. Most surgeries are performed minimally invasively, laparoscopically, which reduces postoperative complications and accelerates the recovery of patients. State-of the-art surgical methods are also routinely performed, such as HIPEC, liver and lung resection. In addition to radical surgeries, palliative surgery is also performed in order to alleviate the symptoms thus improving patients’ quality of life. 

Recently, the Serbian National Training Programme for minimally invasive colorectal surgery (LapSerb, Belgrade, Serbia) was initiated in Serbia with the aim of implementing laparoscopic CRC surgery. Until 2023, 1456 laparoscopic colectomies were performed by 24 surgeons and the program focused on knowledge exchange, workshops, live surgeries and reviewing unedited recordings. LapSerb was successfully and safely established across the country. Short-term clinical outcomes of overall mortality of 1.1% and R0 resections in 97.8% of malignant colectomies were considered comparable and acceptable [33].

#### 3.4.5. Diagnostic Molecular Testing

The Pharmacogenomics Service was established at IORS within the Laboratory for Molecular Genetics in 2008. This service was developed in response to the introduction of anti-EGFR monoclonal antibodies (mAbs), first cetuximab (Erbitux^®^, ImClone/Merck/Bristol-Myers-Squibb, NY, NY, USA) and then panitumumab (Vectibix^®^, Amgen, Thousand Oaks, CA, USA). The task of the service was to fully follow the main principle of pharmacogenetics, which is to determine the adequate drug and dosage for every patient according to genetic characteristics. Until June 2024, a total of 6369 patients underwent testing for somatic mutations in the *KRAS* and/or *NRAS* genes. The individuals tested included 3993 males and 2366 females (65 age median). In 0.16% of patients, the analysis was unsuccessful due to the inadequate quality of the isolated DNA material. Patient characteristics and mutation distribution are presented in Table 1. 

The recommendations for testing have changed over time as knowledge about the effectiveness of these agents has spread. Following the world and European guidelines at IORS, initially, *KRAS* gene exon 2 mutations were the only one analyzed [34]. From 2013, the scope of analysis expanded to include *KRAS* and *NRAS* exons 2, 3, and 4. Today, analyses of *KRAS* and *NRAS* exons 2, 3, and 4 and *BRAF* mutations are recommended at mCRC diagnosis for all patients, while *RAS* testing is obligatory prior toanti-EGFR mAbs treatment [35]. 

**Figure 4:**
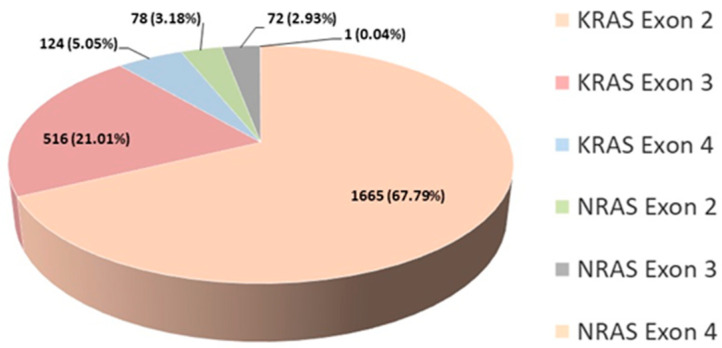
Percentage of detected mutations for 2456 patients tested with the AmoyDx *KRAS/NRAS* Mutations Detection Kit.

According to our findings, 50.43% of all tested patients had tumors that harbored a mutation in the *KRAS* or *NRAS* gene which is in accordance with literature data. The percentage of detected mutations per exon is shown for 2456 patients (Figure 4). The analysis for these patients was consistently conducted using the same kit (AmoyDx^®^ KRAS/NRAS Mutations Detection Kit, Singapore, Singapore), covering all three exons in both genes. As we expected, the highest percentage of mutations was found in *KRAS* exon 2. The total percentage of patients whose tumors had a mutation in the *NRAS* gene is 6.15%, which corresponds to data in the literature (Figure 4).

At IORS, 304 mCRC patients have been analyzed for the presence of *BRAF* mutations. Out of these, 29 patients, or 9.54%, were found to have tumors with a mutation, in agreement with the literature (8–12%) [36]. Currently, this testing is conducted in Serbia only for patients with *RAS* wild type tumors and upon the specific request of the clinician. The literature data suggests that 30% to 40% of CRC carried a *KRAS* mutation, while up to 10% of presented with an *NRAS* mutation. Together, this amounts to 50%, and according to recent research, this may be as high as 60% [37,38]. 

Since 2008, the annual number of patients with mCRC who have been tested on IORS has steadily increased until 2020, when the COVID-19 pandemic hit. The global situation has impacted the healthcare system in Serbia, resulting in a decrease in the number of patients visiting doctors for reasons other than COVID-19 infection, which in turn affected the work of the Pharmacogenomics Service [39,40]. 

**Figure 5:**
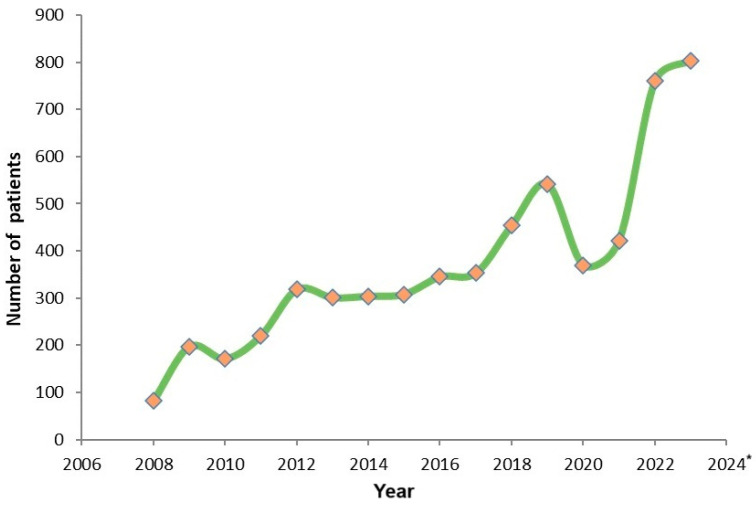
Number of patients tested for of mutations in the *KRAS* and/or *NRAS* gene at IORS in the period 2008–2024. since the establishment of the Pharmacogenomics Service. * Analyses presented for 2024. Include the period 1 January–30 June 2024.

Starting in 2022, there was a greater than expected increase in the number of patients undergoing testing, partly due to the decision by the Republic Health Insurance Fund of Serbia to reimburse anti-EGFR mAbs for use in the first line in mCRC. The previous protocol allowed their use in third-line treatment only. In part, this increase is also due to the decrease in the number of patients who visited IORS during the COVID-19 pandemic (Figure 5) which correlates well with the detected decrease in incidence of CRC in 2020 (Figure 1a).

## 4. Liquid Biopsy Applications

Liquid biopsies are in the limelight as a minimally invasive source of biological material able to provide information to clinicians on the molecular composition of the primary and metastatic tumors at every stage of the clinical care pathway [41]. Circulating cell-free DNA (cfDNA), although containing scarce amounts of circulating tumor DNA (ctDNA), is useful to detect mutations and small insertions and deletions, copy-number alterations, methylation profiles, fragment size profiles or nucleosome-protected fragments reflective of the tissue of origin [42]. Circulating tumor cells, exosomes, tumor educated platelets and circulating RNAs have also shown promise as cancer biomarkers.

The concentration of cfDNA in blood plasma is generally positively correlated with the tumor stage; however, due to high technical variation related to methods used in the pre-analytical stages (blood processing, transport, storage, cfDNA extraction) and high inter-individual physiological variation (anatomical site of the tumor, comorbidities, ongoing therapy, inflammation, presence of cachexia, etc.), it is not a reliable biomarker. Targeted LB assays are more specific and sufficiently sensitive to detect cancer biomarkers. Several assays based on quantitative or digital PCR or NGS can be used to detect variants to direct systemic therapies in a tumor-informed or tumor-agnostic manner. Genes recommended by the ESMO Precision Medicine Working Group for testing in LBs when tumor tissue is unavailable or for time-critical applications, include *BRAF* (for V600E mutation), *NTRK* 1/2/3 fusions, *KRAS/NRAS* mutations (exon 2, 3, 4), *ERBB2* amplification, *EGFR-ECD* (S492, G465, S464, V441 mutations in the extracellular domain), along with MSI-H and TMB score [43]. In the context of therapy selection, clinical trials are being designed to include ctDNA to inform the decision of anti-EGFR rechallenge. The CHRONOS trial has demonstrated a clinical advantage of rechallenge when no prior evidence of *RAS*, *EGFR-ECD*, and *BRAF* mutations in ctDNA exists [44]. Thus far, NGS-based assay FoundationOne Liquid CDx (Foundation Medicine, Inc., Boston, MA, USA) testing a panel of genes is the only cfDNA assay that had received pre-market approval from FDA in 2023 (Premarket Approval (PMA) (fda.gov)) for the detection of BRAF V600E alteration in patients with metastatic CRC from blood plasma. 

Emerging applications include using methylation profiles or fragmentomics. DNA methylation based FDA-approved LB assays for CRC early detection are Epi proColon^®^ (Epigenomics AG, Berlin, Germany) and RealTime mS9 CRC Assay (Abbott Laboratories Chicago, IL, USA) to detect *SEPT9* promoter methylation, and Cologuard^®^ (Exact Sciences Co., Madison, WI, USA) targeting changes in methylation of *BMP3* and *NDRG4* promoters and seven *KRAS* gene point mutations [45]. Ongoing trials are examining the clinical utility of methylation-based multi-cancer early detection strategies. However, according to 2024. NCCN Guidelines for colon and rectal cancer, there is not sufficient level of evidence to recommend individual genes or multi-gene testing using ctDNA assays, outside clinical trials. Similarly, at present, molecular diagnostics from liquid biopsies in CRC is not implemented as the standard of care at the IORS. Pilot clinical programs and scientific projects are being considered for 2025. 

Nevertheless, at IORS, we are engaged in several avenues of translational research using liquid biopsies. Ongoing collection of plasma and FFPE tumor material is organized through the EU Horizon STEPUPIORS project with the aim of establishing the first rectal cancer biobank in Serbia to facilitate ongoing and future translational research. The TRACEPIGEN project which received funding from the Serbian Science Fund implemented a longitudinal study prospectively collecting plasma samples from patients with advanced CRC during systemic therapy, aimed to gain understanding of the genetic and epigenetic evolution of CRC. 

### Significance of Extracellular Vesicles Research in CRC

Extracellular vesicles (EV) enable intercellular communication, and the most studied types are small EVs, up to 120 nm in diameter, often called “exosomes” [46]. While studying the composition of EVs and monitoring glycosylation patterns, various vesicular non-coding RNAs, proteins, metabolites have shown potential as diagnostic and prognostic biomarkers in CRC [47,48,49]. The therapeutic potential of small EVs is under investigation as they might be used as carriers of various non-coding RNAs and chemotherapeutics thanks to their unique properties (size, stability in circulation, encapsulated content, ability to pass through the membrane) [46,50].

In Serbia, an EV-related scientific community has emerged in 2022, with the formation of the Serbian Society for Extracellular Vesicles. The first studies on EVs in CRC are under way within STEPUPIORS, evaluating their potential in profiling the response to neoadjuvant CRT in LARC from sequential plasma samples of IORS patients. Studies involving their use as diagnostic and therapeutic assets are still lacking. 

## 5. Omics Analyses 

### 5.1. Transcriptomics, Genomics and Proteomics

The establishment of the whole genome sequencing (WGS) method led to the discovery of driver mutations and genes in CRC. Single nucleotide variances (SNVs), structural variances (SVs), copy number alterations (CNAs), and small insertion-deletions (indels) are the most common genetic variations discovered in CRC [51]. It was shown that genomic landscapes could be used in addition to clinicopathological features, enabling a more precise definition of the disease subgroup, a personalized approach to treatment and early diagnosis [52]. Genomic studies highlighted mutations that correlate with disease progression, invasiveness, and CRC pathogenesis [53]. Transcriptome analysis, improved understanding of the transcriptional activity of CRC enabling classification of CRC into prognostic subtypes (CRPSs) [54]. A multi-omics approach enables high-throughput insight into the CRC molecular profile [55].

Tumor mutations showed correlation with transcriptomic profile, indicating differences in gene expression pattern between various subtypes of CRC as well as normal and CRC tissue. The state-of-the-art CRC gene expression pattern is Consensus Molecular Subtypes (CMS), which is used for the classification of tumors. CRC prognostic subtypes (CRPSs) predict overall survival (OS), regression-free survival (RFS), and survival after recurrence [54]. Single-cell RNAseq enabled immune landscape profiling of different tumor regions and their association with CRC patient prognosis [56]. Furthermore, the development of proteomics allowed phenotypic profiling and discovering protein level prognostic biomarkers of disease, thus enabling personalized management of CRC [57]. High-throughput proteomic analysis conducted on rectal cancer tissue in our population indicated high predictive potential of treatment response by highlighting proteins encoded by genes *SMPDL3A*, *PCTP*, *LGMN*, *SYNJ2*, *NHLRC3*, *GLB1* and *RAB43* as markers of unfavorable response and *RPA2*, *SARNP*, *PCBP2*, *SF3B2*, *HNRNPF*, *RBBP4*, *MAGOHB*, *DUT*, *ERG28* and *BUB3* of favorable response [58]. Genomic and transcriptomic studies conducted in the Serbian science community mainly focus on single gene analysis to improve clinical diagnostic and patient monitoring. Some of the many published results include detection of *KRAS* and *BRAF* mutations in Serbian population [34,59,60] and development of less invasive assay for detection of those mutations by using ddPCR [61]. The discovery of predictive biomarkers on mRNA level by using in silico methods indicated the importance of inflammatory response in treatment outcome while *IDO1* gene expression level shown to have predictive potential [62]. Detection of SNPs using cost-effective methods in MTHFR gene highlighted their role in CRC risk [63]. Serum level of circulating miR-93-5p for patients diagnosed with CRC and liver metastases shown to be a prognostic factor for early disease recurrence [64]. Analysis of transcript *CD81-215*, *SMAD4-209*, *SMAD4-213*, and *SMAD7* 3′UTR variants indicated their importance in CRC and malignant transformation [65,66,67].

### 5.2. Radiomics

The term “radiomics” was first introduced over a decade ago [68], marking the fusion of radiology with omics sciences. As imaging technologies advanced and image resolution improved, it became obvious that medical images contain far more information than what was being utilized in routine clinical practice. This insight, along with the rise of big data analytics and machine learning, paved the way for the development of radiomics. The power of radiomics is in its potential to perform unselective computational texture analysis, revealing hidden prognostic data which was unexploited by visual or computational evaluations of specific structures. Radiomics is evolving rapidly, extracting a very large number of first-, second- and higher-order quantitative statistical texture features from medical images, such as CT, MRI or PET scans. Using sophisticated machine learning (ML) bioinformatics tools, radiomics data and other patient data are combined to develop models, mostly to prognosticate binary endpoints such as therapy response or disease outcome. This approach enables personalized medicine, thus improving patient survival [69]. 

The comprehensiveness of radiomics is its primary advantage because its analyses outperform more traditional fractal and texture analyses which calculate only several features [70]. However, using such a large number of features is also a drawback, because high dimensionality can cause overfitting, where the model becomes complex and performs satisfactorily on training data but worse on unseen data. Additionally, high dimensionality can obscure the most relevant features, making it difficult to derive meaningful insights from the data. To address this problem, feature selection methods like the Least Absolute Shrinkage and Selection Operator (LASSO) are used to lower the number of features included in the final model. Subsequently, machine learning classifiers such as Support Vector Machines (SVM), random forests and neural networks are utilized to perform the classification against a binary outcome. By combining feature selection methods with machine learning algorithms, radiomics can overcome the challenges of high dimensionality, leading to more accurate and generalizable predictive models.

Radiomics technology is gaining traction in Serbia, with four studies listed on PubMed, all originating from our laboratory. Radiomics has been applied as a tool to enhance the prediction of chemoradiotherapy response of rectal cancer [71], breast cancer metastasis [72], chemotherapy response of osteosarcoma [73] and radiotherapy response of meningioma [74]. A unique approach was undertaken in breast cancer prognosis, where radiomics analysis characteristic fo3D scans was applied to 2D histopathology slides, to assess tumor heterogeneity at the histological level [72].

The study on LARC patients was conducted to produce a machine learning model that integrates radiomics from pretreatment MRI 3D T2W contrast sequence scans with clinical data to predict the response nCRT. Tumor characteristics were evaluated by calculating 1862 radiomics features, with feature selection performed using LASSO and multivariate regression. The models demonstrated a moderately advantageous impact of increased dimensionality, with low- and high-dimensional models, including 93 and 1862 radiomics features, respectively, achieving predictive AUCs of 0.86 and 0.90. Both models that included radiomics features were of higher performance than the CP-only model (AUC = 0.80), which had been considered as a cornerstone of predictive success without radiomics. These findings suggest that combining MRI radiomics with clinical features could serve as an improved predictor of nCRT response in LARC, aiding clinicians in personalizing treatment [71]. 

### 5.3. Metabolomics

Metabolomics represents the status of all metabolites within the body and is highly correlated to disease phenotype. It can provide clinically useful biomarkers for diagnostics and patient stratification in CRC. Patient metabolomics profiles can distinguish healthy controls from CRC patients, categorize different stages of disease, and predict early or late onset disease [75]. Also, it is helpful in the discovery of new therapeutic targets and monitoring the activity of therapeutics. Holowatyj et al. revealed that patients with early and late-onset CRC can be differentiated by metabolic profiles, with more pronounced metabolic dysregulation present in patients with late-onsetCRC [76]. Metabolomic studies of Geijson et al. and Liu et al. showed that metabolomic profiles of patients with different stages of CRC are correlated with specific regulatory mechanisms of metabolism [77,78]. Many studies indicate that metabolomic studies are promising tool for screening purposes identifying potentially important metabolic features linked with risk of developing CRC risk [79,80].

In Serbia, metabolomics studies are still underutilized in clinical CRC practice but represent a promising tool for identifying CRC biomarkers with high sensitivity and specificity. As part of the STEPUPIORS project, initial retrospective and prospective metabolomics studies are under way to profile the response to neoadjuvant CRT in LARC from sequential liquid biopsy samples of patients undergoing treatment at IORS. The first results are expected in 2025. 

### 5.4. Fragmentomics

The analysis of fragmentation patterns in cell-free DNA (cfDNA), referred to as fragmentomics, has proven to be a useful approach for extracting information from liquid biopsies without requiring mutational data [81]. Tumor tissue releases DNA (ctDNA) that is different in size compared to healthy cells, which also displays both genetic and epigenetic changes derived from its originating cells, including fragment endpoints, specific motifs and nucleosome characteristics. Concerning CRC, a few fragmentomics-based assay models have been proposed, which managed to distinguish CRC patients from healthy controls, enabled early-stage diagnosis, risk detection and broad patient benefits [82,83]. As it represents an emerging field in research of cancer biomarkers, detection of cancer in early stages and diagnosis, the first fragmentomics study is under way to profile the response to neoadjuvant CRT in LARC from sequential liquid biopsy samples of patients undergoing treatment at IORS within the STEPUPIORS Horizon Europe project.

## 6. Discussion

Although significant improvements in CRC management have occurred globally in recent years, physicians and the scientific community in Serbia are still facing many challenges, similar to global trends. Current concerns as low adherence to screening, the high incidence of young-onset CRC, the growing elderly and limited survival of patient with metastatic disease require a strategic approach leading to population-based systemic solutions as seen in some European countries [84]. Approaches to counseling of genetic and fertility nature in young adults are currently without proper systemic care at the national level. All these issues induced responses from the Serbian medical and scientific community in accelerating programs for prevention and earlier detection, increasing the availability of treatment options and modern techniques as well as scientific research focused on our population. 

From the clinical point of view, progress seen in the past two decades in Serbia is encompassing implementation of minimally invasive surgeries and state-of-the art surgical methods, adherence to modern radiotherapy techniques, evolution of systemic treatment and implement of genetic counseling that allows defining patient subpopulations with CRC induced by germline pathogenic variants. From the research part, huge efforts are needed to establish a sequential relationship between changes in biomarkers with disease progression prediction of treatment outcomes. Integration of clinical data with multidimensional imaging and biological multiomics information collected from patient cohorts will be the focus of new lines of research in Serbia. Liquid biopsy approaches and modern, sensitive techniques might prove to be essential not only in the management of metastatic disease, but also early and locally advanced CRC.

The ongoing efforts include a global approach towards decreasing the inequity in cancer research which can lead to better treatment strategies and steer societal impact in countries with limited research and health resources, as Serbia. This might only be addressed by progressing beyond descriptions of cancer outcome differences towards a systematic, non-profit, long-term strategic initiatives and active collaborations among all relevant cancer stakeholders and existing social structures, with a focus on improving equitable access to healthcare services, broadening clinical research, working on resolving structural limitation, and increasing awareness that might lead to tangible and time-precise measures toward overall better outcomes. This is in line with relevant global initiatives as the ones recently launched by ESMO and ASCO [85,86]. Studies focusing on utilization patterns and medical cost in oncology are needed in Serbia and the Balkan region, in order to advance evidence-based policy changes with adequate return on investments. 

## 7. Specific Challenges and Future Perspectives

Key challenges for reducing CRC incidence and mortality in Serbia remain to be addressed in the coming years:

- specific national diet rich in fat and processed foods and depleted in fiber
- high incidence of obesity and sedentary lifestyle
- low uptake of national screening/need for increased awareness on the benefits of prevention and early detection
- improve diagnostic techniques and increase availability of medical equipment
- expanding the molecular testing of metastatic CRC (including HER2 amplification, NTRK, MSI)
- improve drug availability and reimbursement
- improve minimally invasive surgical techniques and postoperative follow-up
- introduce liquid-biopsy based molecular diagnostic approaches and follow-up
- strengthen national heath technology analysis approaches
- provide continuous training for healthcare workers and
- increase the number of scientific projects and funding dedicated to CRC research through interdisciplinary collaborative efforts.

Main challenges in risk reduction need to be addressed through promoting healthy lifestyle choices and encouraging adherence to screening at a primary healthcare level. Proper education of the primary care physician has to be improved, particularly in the light of the recent trends in rising in the early-onset CRC. In addition, public appearances and the dissemination of information at a national level by oncology experts must be part of the effort to address the problem of late-stage diagnosis. Individual treatment approaches can be improved by integrating expanded molecular testing in everyday clinical practice, which has to be followed by improved accessibility and availability of novel anti-cancer drugs. Incorporating liquid biopsies in monitoring MRD, as well to monitor treatment effectiveness and drug re-challenge options, need to be the focus of future efforts. This must align with ongoing capacity building for healthcare workers at all levels, while promoting the success rate of scientific project applications, enhancing international collaboration, and increasing the international visibility of the Serbian oncology society.

## 8. Conclusions

The combined effort of clinical and research oncology professionals is of paramount importance in achieving the common aim, to lower the numbers of newly diagnosed patients and improving the quality of life and survival of the patients with CRC. Progress in personalized treatment of CRC in the past two decades in Serbia has been marked by multidisciplinary work. Although there are many future challenges, multidisciplinary interventions and a personalized approach to every patient will be the cornerstone for facing them.

## Figures and Tables

**Table 1:** Characteristics of patients and the distribution of mutations by exons for both genes of all subjects tested at IORS from June 2008 to June 2024.

			KRAS Mutations				NRAS Mutations			
	Number (%)	Age Range (Median)	WT	Exon 2	Exon 3	Exon 4	WT	Exon 2	Exon 3	Exon 4
All patients	6359 (100)	18–97 (65)	3316	2772	120	166	3425	89	97	2
Male	3993 (62.8)	18–97 (65)	2146	1686	69	102	2145	59	66	1
Female	2366 (37.2)	18–90 (65)	1170	1086	51	64	1280	30	31	1

## Data Availability

The mass spectrometry proteomics data have been deposited to the ProteomeXchange Consortium via the PRIDE partner repository (https://www.ebi.ac.uk/pride/, accessed on 3 October 2024) with the dataset identifier PXD040451. The radiomics data are openly available in Zenodo, at doi: 10.5281/zenodo.8379940. Other data are not publicly available due to ethics restrictions as their containing information might compromise the privacy of patients.
